# Human breast cancer cell-mediated bone collagen degradation requires plasminogen activation and matrix metalloproteinase activity

**DOI:** 10.1186/1475-2867-5-1

**Published:** 2005-02-08

**Authors:** Hayley Morgan, Peter A Hill

**Affiliations:** 1 Department of Craniofacial Biology and Orthodontics, King's College London, Floor 22, Guy's Tower, Guy's Hospital, London, SE1 9RT UK

## Abstract

**Background:**

Breast cancer cells frequently metastasize to the skeleton and induce extensive bone destruction. Cancer cells produce proteinases, including matrix metalloproteinases (MMPs) and the plasminogen activator system (PAS) which promote invasion of extracellular matrices, but whether these proteinases degrade bone matrix is unclear. To characterize the role that breast cancer cell proteinases play in bone degradation we compared the effects of three human breast cancer cell lines, MDA-MB-231, ZR-75-1 and MCF-7 with those of a normal breast epithelial cell line, HME. The cell lines were cultured atop radiolabelled matrices of either mineralized or non-mineralized bone or type I collagen, the principal organic constituent of bone.

**Results:**

The 3 breast cancer cell lines all produced significant degradation of the 3 collagenous extracellular matrices (ECMs) whilst the normal breast cell line was without effect. Breast cancer cells displayed an absolute requirement for serum to dissolve collagen. Degradation of collagen was abolished in plasminogen-depleted serum and could be restored by the addition of exogenous plasminogen. Localization of plasmin activity to the cell surface was critical for the degradation process as aprotinin, but not α_2 _antiplasmin, prevented collagen dissolution. During ECM degradation breast cancer cell lines expressed urokinase-type plasminogen activator (u-PA) and uPA receptor, and MMPs-1, -3, -9,-13, and -14. The normal breast epithelial cell line expressed low levels of MMPs-1, and -3, uPA and uPA receptor. Inhibitors of both the PAS (aprotinin and PA inhibitor-1) and MMPs (CT1166 and tisue inhibitor of metalloproteinase) blocked collagen degradation, demonstrating the requirement of both plasminogen activation and MMP activity for degradation. The activation of MMP-13 in human breast cancer cells was prevented by plasminogen activator inhibitor-1 but not by tissue inhibitor of metalloproteinase-1, suggesting that plasmin activates MMP-13 directly.

**Conclusions:**

These data demonstrate that breast cancer cells dissolve type I collagen and that there is an absolute requirement for plasminogen activation and MMP activity in the degradation process.

## Background

Breast cancer is the most frequent cancer in the female population of industrialized countries. Metastasis of breast cancer cells to the skeleton occur in >70% of patients with progressive disease, resulting in debilitating symptoms such as severe bone pain, fractures, hypercalcaemia and spinal cord or nerve compressions due to extensive bone loss and tumour cell growth and expansion. Such bone loss occurs as a result of increased bone matrix resorption but the mechanisms by which cancer cells mediate this increased degradation have not been fully elucidated. Obviously, tumour expansion in bone requires the removal of the extracellular matrix (ECM) that is particularly abundant in bone. Cancer cells express matrix metalloproteinases (MMPs) and the plasminogen activator system (PAS) [1-3] and their levels of expression increase with progression of the tumour.

The matrix metalloproteinases (MMPs) constitute a large family of structurally related matrix degrading proteases that have pivotal roles in development, tissue remodelling, and cancer [4-6]. The gene family of MMPs includes the interstitial collagenases (MMPs-1 and -13), gelatinase A (MMP-2), gelatinase B (MMP-9), the stromelysins (MMPs-3, 10 and 11) and the membrane type-matrix metalloproteinases (MT-MMPs 14,15,16,17, 24 and 25) [6]. The MMPs have the combined ability to degrade the major components of the ECM including type I collagen, the principal organic constituent of bone [4].

The PAS comprises: tissue-type plasminogen activator (t-PA) and urokinase-type plasminogen activator (u-PA), their inhibitors, and receptors. T-PA is thought to be more important in fibrinolysis, due to its fibrin binding capacity, whilst u-PA, especially when it is bound to its specific cell surface receptor (u-PAR), is thought to be involved in tissue remodelling and cell migration processes [7]. Whereas uPA alone recognizes a narrow range of substrates, the enzyme can catalyze the conversion of the circulating zymogen, plasminogen to plasmin. Plasmin, in turn, is a broad- spectrum proteinase that can directly degrade multiple ECM targets and can also cooperate with other ECM-degrading enzymes including members of the MMP gene family. Regulation of the PA/plasmin system is achieved mainly via plasminogen activator inhibitor (PAI) type-1 and type-2 and by agents that stimulate bone resorption, e.g. parathyroid hormone (PTH) and interleukin (IL)-1 [8].

To date, emphasis has focused on the ability of breast cancer cells to stimulate the formation and activity of osteoclasts, the cell primarily responsible for bone resorption under physiological conditions. The ability of osteoclasts to degrade bone lies in their ability to secrete protons and specialized collagenolytic proteinases, the cysteine proteinases in the acidic microenvironment that underlies osteoclasts during bone resorption [9].

Experimental studies showing that increased expression of MMPs and the PAS is associated with increased cellular invasion *in vivo *support the idea that they play an important role in metastasis of tumour cells [10,11]. Obviously tumour expansion in bone necessitates the removal of the ECM that is particularly abundant and resistant to degradation. Synthetic inhibitors of MMPs have been developed and two recent reports on their use on *in vivo *breast cancer metastasis to bone show promise when given as a preventive treatment to mice [12,13]. However, the role of MMPs and the PAS in mediating breast tumour bone collagen dissolution has not been addressed.

We have therefore assessed the ability of three human breast cancer cell lines, MDA-MB-231 (MDA-231), ZR-75-1 and MCF-7 to degrade bone collagen *in vitro *using matrix degradation assays and compared their effects with those of a normal breast epithelial cell line, HME. We correlated the degradation activity of the breast cancer cells with their expression of MMPs and the PAS and we assessed the ability of group-selective proteinase inhibitors to prevent degradation of the organic aspect of bone by breast cancer cells.

## Results

### Type I collagen degradation

**Figure 1:**
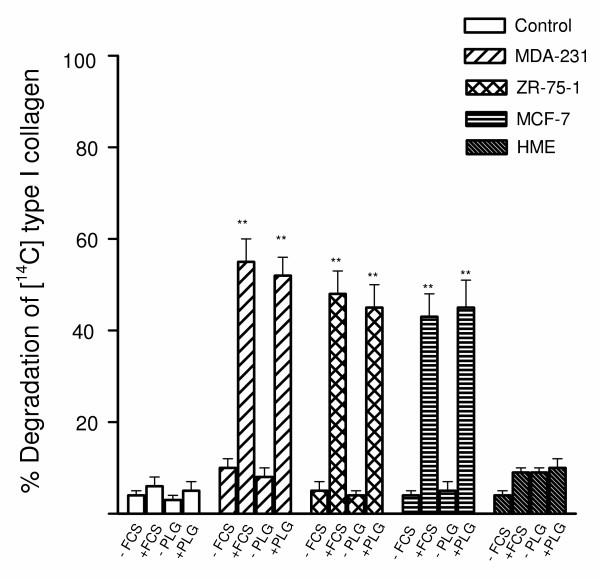
**Degradation of ^14^C-labelled type I collagen films by breast cancer cells. **Breast cancer cells (10^5 ^cells/well) stimulated with TGFβ (10^-10 ^M) were cultured for 24 h on ^14^C-labelled type I collagen under the following conditions: serum-free conditions; presence of 10% serum; 10% plasminogen (Plg)-depleted serum; serum-free medium supplemented with 2 μg/ml of human Plg. After 24-h incubation, collagen degradation was measured as described in Materials and methods. 8–800 mU (Ploug units) of pure human uPA in 1 ml of serum-free medium with or without 2 ug/ml of plasminogen, incubated as a control in parallel wells in the absence of cells, released 2–4% of the total radioactivity. This experiment was repeated twice. The results are expressed as percentage release of ^14^C. Each bar is the mean ± S.E.M of six wells. The stimulatory effects of plasminogen and TGFβ on breast cancer cell mediated ^14^C release were statistically significant ***P < 0.001 compared with the unstimulated controls and the HME cells.

Bone degradation involves an initial phase of removal of the unmineralized type I collagenous layer followed by degradation of the mineralized matrix which also comprises type I collagen. The fibrillar integrity of the collagen layer was confirmed by incubation with collagenase that degraded the film whilst the collagen fibres were resistant to degradation by both trypsin and plasmin (data not shown). When the breast cancer cells (MDA-231, ZR-75-1 or MCF-7) were stimulated with TGFβ(10^-10^M) and cultured in the presence of 10% FCS the cancer cells induced significant degradation of type I collagen (range 70–80%), whereas minimal degradation was observed in the absence of serum (Fig. 1). To investigate the possible role of the PAS in collagen breakdown by breast cancer cells, plasminogen was depleted from FCS by lysine-Sepharose chromatography [14]. Interestingly, the depletion of plasminogen from serum also completely blocked breast cancer cell mediated collagen dissolution, implicating the PAS in breast cancer-mediated collagen degradation (Fig. 1). In accordance with this finding, the breast cancer cells degraded collagen under serum-free conditions only when supplemented by exogenous plasminogen (Fig. 1). The TGFβ-stimulated normal breast cell line, HME cultured in the presence of 10 % FCS demonstrated low type I collagenase activity (Fig. 1).

The ability of the cancer cells to degrade the type I collagen is consistent with the expression of proteolytic activity. However, the midpoint melting temperature of reconstituted firbrillar type I collagen (47°C) is lower than that of authentic type I collagen in tissues (55°C to 60°C) [4]. Because the proteinase resistance of type I collagen can be compromised at temperatures within 10°C of that at which the helix reversibly unfolds [4], reconstituted fibrillar collagen may provide a less resistant substrate to proteolytic activity. Hence to determine whether breast cancer cell mediated degradation of type I collagen could be extended to a more physiological system, cancer cells were cultured on bone matrix as described below.

### Bone matrix degradation by breast cancer cells

The described sequential gene expression of differentiating osteoblasts [15] was verified in MC3T3-E1 cell cultures so that non-mineralized matrix production was prepared after collagen production had commenced but before mineralization started. Mineralization of the matrices was confirmed by von Kossa staining (not shown).

**Figure 2:**
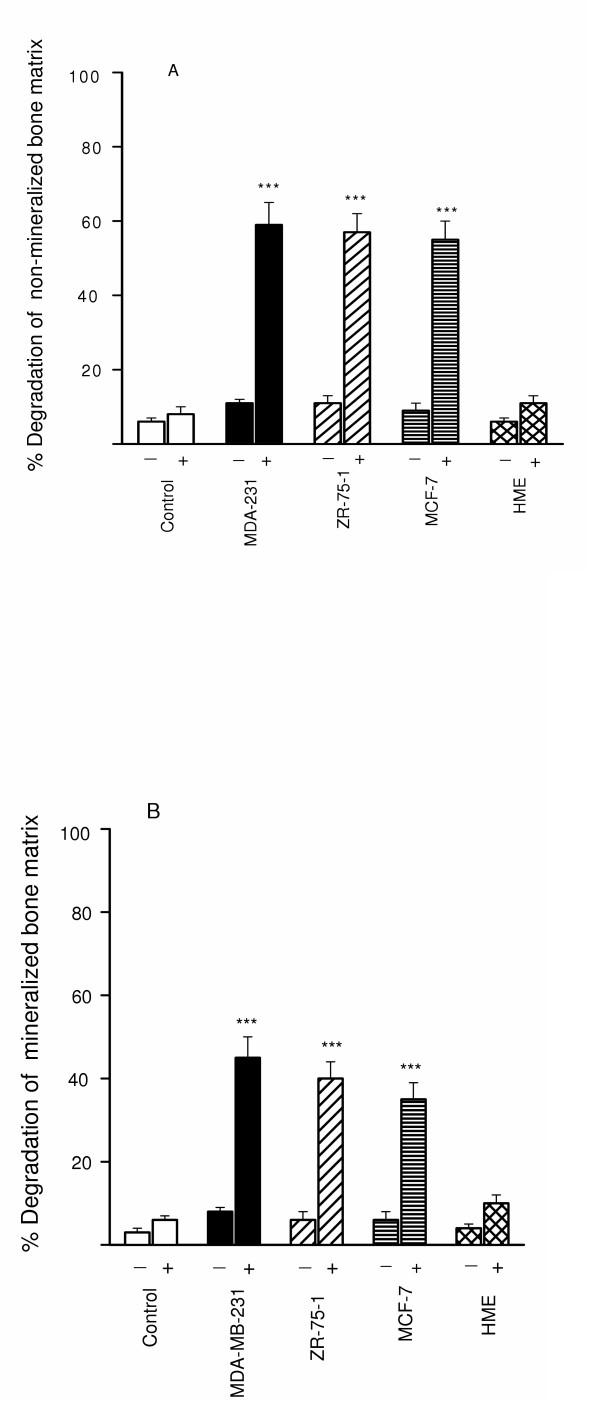
**Degradation of ^3^H-non-mineralized (A) and ^3^H-mineralized (B) bone matrix by breast cancer cells**. Breast cancer cells (10^5 ^cells/well) stimulated with TGFβ (10^-10 ^M) were cultured for 24 h on ^3^H-labelled extracellular matrices in the presence (+) and absence (-) of 2 μg/ml of human plasminogen. After 24-h incubation, bone matrix degradation was measured as described in Materials and methods. 8–800 mU (Ploug units) of pure human uPA in 1 ml of serum-free medium with or without 2 μg/ml of plasminogen, incubated as a control in parallel wells in the absence of cells, released 3–4% of the total radioactivity. This experiment was repeated twice. The results are expressed as percentage release of ^3^H labelled bone matrix. Each bar is the mean ± S.E.M. of six wells. The stimulatory effects of TGFβ on breast cancer cell mediated ^3^H release were statistically significant ***P < 0.001 compared with the unstimulated controls and the HME cells.

When TGFβ-stimulated breast cancer cell lines were cultured as a monolayer on either non-mineralized (Fig. 2A) or mineralized (Fig. 2B) bone matrix over a 24 h culture period there was significant degradation of the non-mineralized matrix (range 65–75%; Fig. 2A) and to a lesser extent the mineralized matrix (range 40–45%; Fig. 2B) only in the presence of plasminogen. In contrast, the TGFβ-stimulated breast cancer cell lines achieved a minimal amount of degradation of either matrix in the absence of plasminogen (Fig. 2A and 2B). The normal breast cell line, HME achieved a minimal amount of degradation of both matrices (5–15%) in the presence of plasminogen.

### Expression of mRNA for MMPs and the PAS by breast tumour cells

**Figure 3:**
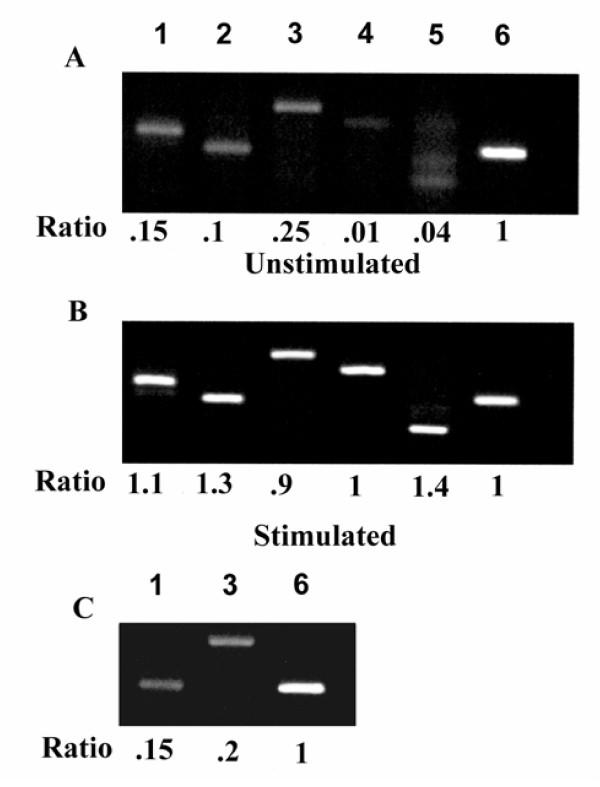
**RT-PCR of MMPs in breast cancer cells. **Breast cancer cells were cultured as described in the Material and methods in the absence (A) and presence (B) of TGFβ (10^-10 ^M). Total RNA was isolated and RT-PCR performed with specific primers for MMPs-1,2,3,7,8,9,10,11,12,13,14,15,16,17. The housekeeping gene GAPDH was used as a positive control. Representative results for MDA-231 cells are shown (A) and (B): Lane 1 MMP-1; lane 2 MMP-13; lane 3 MMP-3; lane 4 MMP-9; lane 5 MMP-14; lane 6 GAPDH. The normal breast cell line HME stimulated with TGFβ is shown in (C). Band intensities were quantified by scanning densitometry and data expressed as a ratio (MMP/G3PDH) of the average optical density (OD) × area. The ratio of the intensity of the MMP mRNA band over the intensity of the G3PDH mRNA was arbitrarily designated as 1.0.

To characterize the profile of MMPs expressed constitutively and upon stimulation with TGFβ, breast cancer cells were cultured on type I collagen. Total RNA was isolated from 24-h cultures and screened by reverse transcription-PCR for MMP-1 through MMP-17. Under these conditions, four secreted MMPs were identified: MMP-1, MMP-3, MMP-9, and MMP-13 and the membrane-anchored MMP, MT1-MMP in all 3 breast cancer cell lines (Fig 3A; MDA-231 cells shown, results similar in all 3 breast cancer cell lines). TGFβ upregulated MMP expression in all 3 cancer cells lines (Fig. 3B; MDA-231 cells shown, results similar in all 3 breast cancer cell lines). The normal epithelial cell line, HME was found to express low levels of MMP-1 and MMP-3 upon stimulation with TGFβ (Fig. 3C)

**Figure 4:**
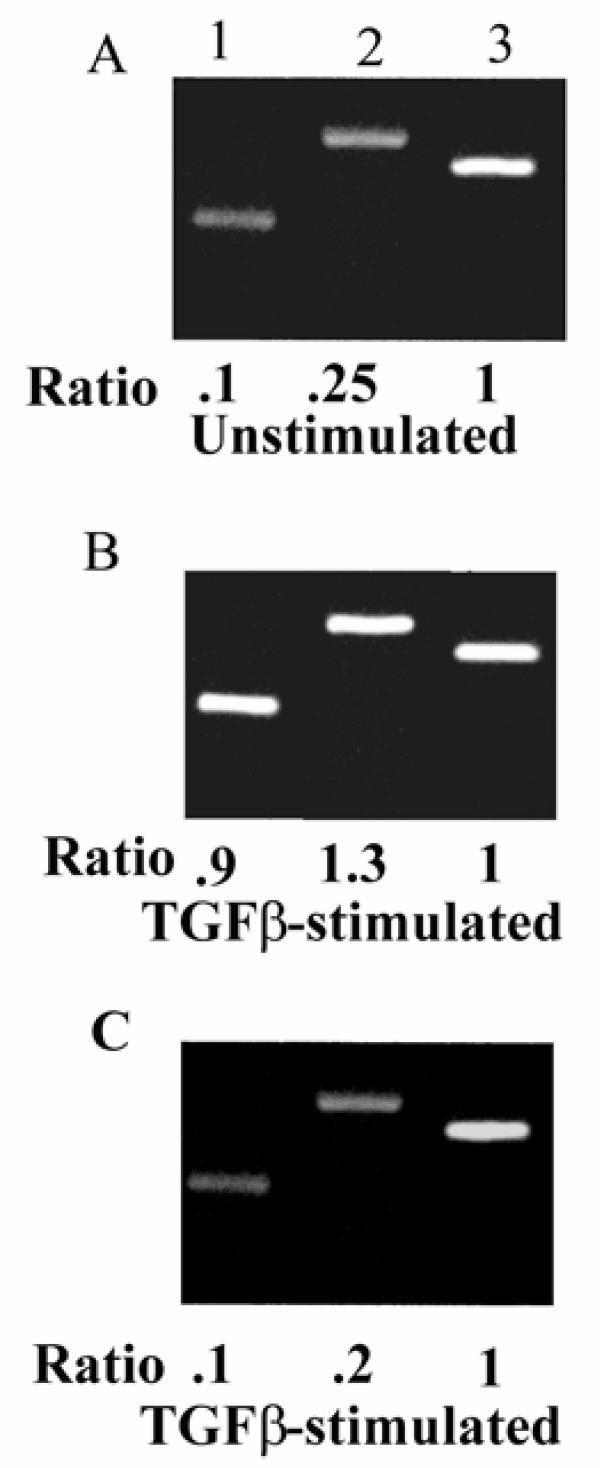
**RT-PCR of uPA and uPAR in breast cancer cells. **ZR-75-1 breast cancer cells were cultured as described in the material and methods in the absence (A) and presence (B) of TGFβ (10^-10 ^M). Total RNA was isolated and RT-PCR performed with specific primers for uPA, tPA and uPAR. The housekeeping gene GAPDH was used as a positive control. Lane 1 uPA; lane 2 uPAR; lane 3 GAPDH. The normal breast cell line HME stimulated with TGFβ is shown in (C). Band intensities were quantified by scanning densitometry and data expressed as a ratio (uPA or uPAR/G3PDH) of the average optical density (OD) × area. The ratio of the intensity of the uPA or uPAR mRNA band over the intensity of the G3PDH mRNA was arbitrarily designated as 1.0.

All 3 breast cancer cell lines expressed u-PA and u-PAR (Fig. 4A; ZR-75-1 cells shown, results similar with all 3 breast cancer cell lines) and upon stimulation with TGFβ there was increased expression of both u-PA and u-PAR by the breast cancer cells (Fig. 4B; ZR-75-1 cells shown, results similar with all 3 breast cancer cell lines.). The intensity of the signal was greater for the breast cancer cells than the normal breast cell line, HME (Fig. 4C).

### MMP and uPA production by breast cancer cells

**Figure 5:**
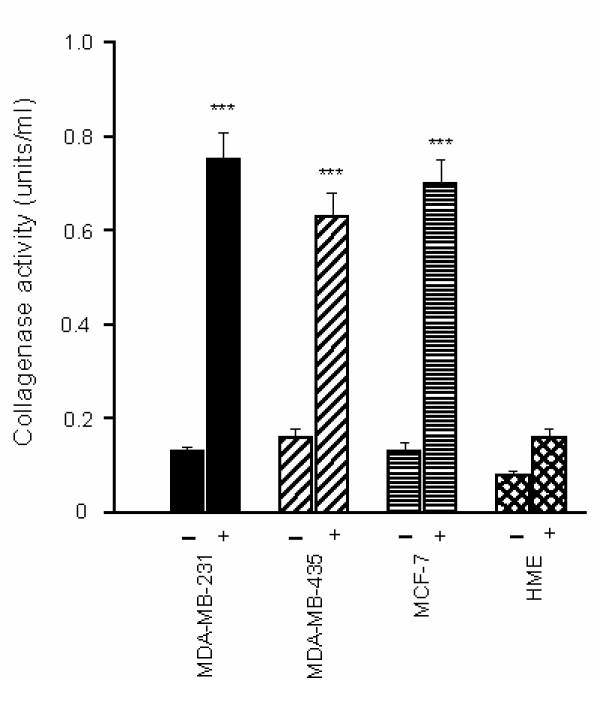
**Collagenase activity in breast cancer conditioned media **Breast cancer cells were cultured for 24 h in serum-free medium in the presence and absence of 2 μg/ml of human plasminogen and TGFβ (10^-10 ^M). Conditioned media were collected and incubated for 4 h with FITC-labelled type I collagen to detect collagenase activity, as described in Material and methods. The data are expressed as means SEM of 4–6 independent experiments, significantly different from control (**, P < 0.01; *** P < 0.001).

To analyze the functional activities of MMPs and PAs expressed in breast cancer cells, collagenase activity in BCCM was measured by the degradation of FITC labelled type I collagen in the presence and absence of plasminogen. TGFβ markedly stimulated collagenase activity only in the presence of plasminogen (Fig. 5). uPA production was high in all cancer cells which degraded bone matrix but not in HME cells (Table 1) that did not degrade bone at all, suggesting that uPA may be necessary to accomplish this task.

**Table 1:** Production of uPA by Breast Cancer Cells

Cells	uPA (U/1 × 10^5 ^cells/24 h)
MDA-231	2.3
ZR-75-1	1.9
MCF-7	1.6
HME	Undetected

uPA was measured by a chromogenic assay (see Materials and methods) in serum-free conditioned medium collected over a 24 h period. Breast cells were cultured in the presence of plasminogen (2 ug/ml) and TGFβ (10^-10 ^M). uPA was not detected in unstimulated breast cancer cells.

### Roles of MMPs and PAS in breast cancer cell mediated bone degradation

**Figure 6:**
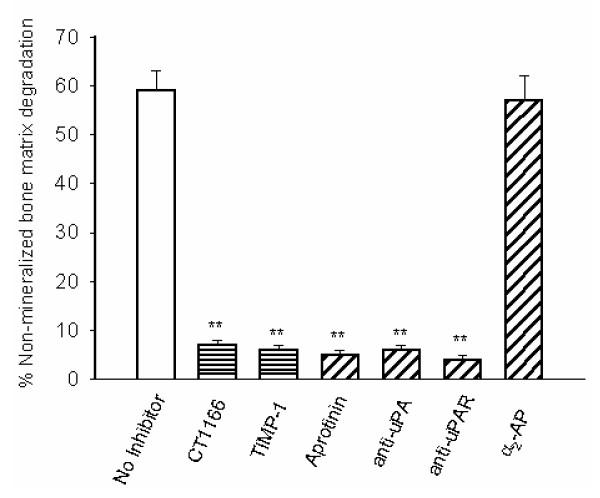
**Effects of MMP and PAS inhibitors on MDA-231 cell mediated degradation of non-mineralized matrix. **MDA-231 breast cancer cells (10^5 ^cells/well) were cultured for 24 h on ^3^H-labelled extracellular matrices in the presence of 2 ug/ml of human plasminogen, TGFβ (10^-10 ^M) with and without CT1166 (10^-5 ^M), TIMP-1 (50 ug/ml), aprotinin (10^-5 ^M), antibodies to human uPA (50 μg/ml) or human uPAR (50 μg/ml). After 24-h incubation, bone matrix degradation was measured as described in Materials and methods. This experiment was repeated twice. The results are expressed as percentage release of degradation of ^3^H labelled bone matrix. Each bar is the mean ± S.E.M. of six wells. The effects of the inhibitors were statistically significant *P < 0.05;***P < 0.001.

**Figure 7:**
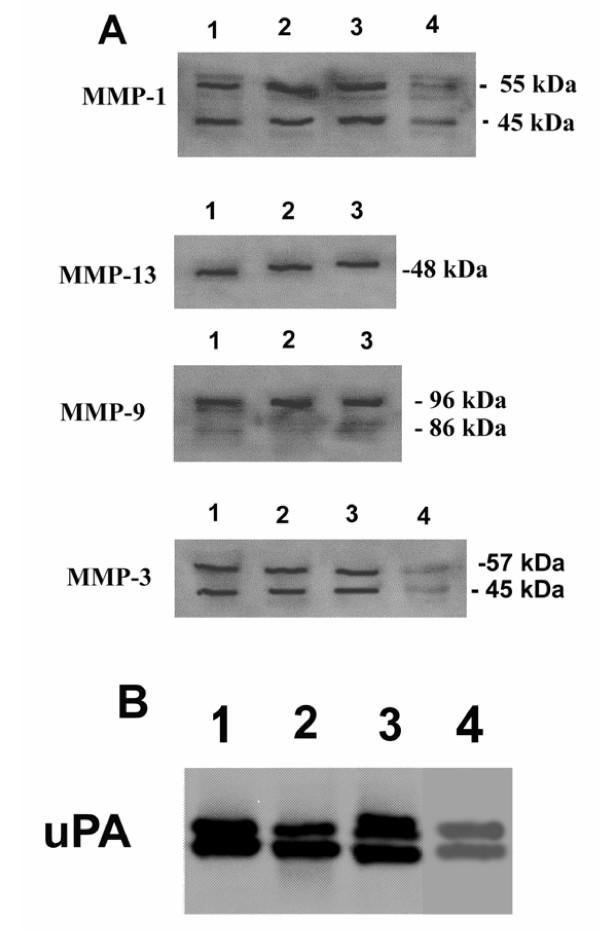
**Immunological characterization of MMPs and uPA in Breast Cancer Cells. **Breast cancer cells (10^5 ^cells/well) stimulated with TGFβ (10^-10 ^M) were cultured for 24 h in serum-free medium in the presence of 2 μg/ml of human plasminogen and CT1166 and aprotinin. Western blot analysis was undertaken as described in the Materials and methods section. Lane 1, MDA-231 cells; lane 2, ZR-75-1cells; lane 3 MCF-7 cells; lane 4, HME cells. Pro- and active forms of collagenase-1 gelatinase-B, and stromelysin-1 and proform of collagenase-3 were detected (A). Pro and active forms of uPA are shown (B).

We examined the effects of inhibitors of MMPs and the PAS on TGFβ-stimulated MDA-231 cell-mediated degradation of non-mineralized bone matrix under serum free conditions supplemented with plasminogen. The MMP inhibitors, CT1166 and TIMP-1 completely prevented TGFβ-stimulated breast cancer cell mediated bone collagen degradation (Fig. 6). When aprotinin, which inhibits both plasmin bound to the cell surface and plasmin in solution, was added, collagen degradation was also completely blocked (Fig. 6). Similar inhibitory effects were seen with function blocking antibodies to uPA or PAI-1 (Fig. 6). In contrast, the serpin α_2_-antiplasmin, which is a poor inhibitor of cell surface bound plasmin but an excellent inhibitor of plasmin in solution, did not prevent collagen degradation (Fig. 6). Since in the absence of cells, plasmin had no collagenolytic activity (see legend to Figures 1 and 2) and none of these inhibitors was cytotoxic, (data not shown), these results showed that bone collagen degradation by human breast cancer cells is dependent upon plasminogen activation and MMP activity. Western blot analysis demonstrated that neither CT1166 nor aprotinin influenced the production of MMPs (Fig. 7A) or uPA (Fig. 7B).

## Discussion

The data presented in this paper clearly demonstrate that human breast carcinoma cell lines have the capacity to degrade the organic aspect of bone matrix *in vitro*, and there is a dependency on the PA system for the cell-mediated collagen degradation. Furthermore, we have shown that plasmin associated with the cell surface is responsible for activating the fibrillar collagenase, MMP-13.

The ECM in our experiments was produced by MC3T3-E1 mouse calvarial-derived cells. These cells display osteoblast-like characteristics, providing a suitable model of osteogenesis analogous to *in vivo *bone formation [17]. The bone nature is evident from the appearance of mineralization, resulting in the production of a solid sheet of mineralized matrix. Our evidence showing that breast cancer cells degrade bone matrix is in agreement with that of Eilon and Mundy [18] who reported that MCF-7 cells were capable of degrading the organic aspect of devitalized murine bone explants *in vitro*. More recently it has been demonstrated that prostate cancer cells and melanoma cells directly degrade mineralized bone matrix and that the degradation was reduced by generalized inhibition of MMP activity [19,20].

Whereas the induction of MMPs in TGFβ stimulated breast cancer cells that are actively engaged in tumour osteolysis has not been examined previously, this growth factor has been reported to increase the expression of MMPs-1, -3 and -9 [21]. In our study TGFβ induced a complex MMP expression profile that included MMPs-1, -3, -9 -13, and -14 as the principle products. Furthermore, MMPs are able to release and activate TGFβ, a very abundant bone matrix-bound factor [22].

The fact that breast cancer cells degraded the non-mineralized bone matrix to a greater extent than the mineralized substrate, suggests that access of the matrix degrading proteinases to the organic phase of the matrix may have been restricted by the mineralized phase.

This investigation has demonstrated a significant difference in the ability of breast cancer cells and normal breast epithelial cells to degrade the organic aspect of bone matrix *in vitro*. Variations in the profile and amounts of proteinases expressed by the different cell lines may be responsible. Since the MMP inhibitors used in this study (CT1166 and TIMP-1) inhibit the activities of all MMPs, except the membrane-type MMPs in the case of TIMP-1, we cannot identify the contribution of individual MMP members to bone matrix degradation. However, our demonstration of a direct requirement of plasmin in the activation of proMMP-13 suggests that this fibrillar collagenase may play a prominent role in the degradative activity of breast cancer cells. However, the possibility of other known MMPs also being involved in plasminogen-dependent collagenolysis by breast cancer cells cannot be excluded. The participation of the membrane-type MMPs such as MMP-14, -15 and -16 is improbable due to the ability of TIMP-1 to effectively block collagen dissolution by breast cancer cells. MMP-2 and – 8 were not expressed in detectable amounts by breast cancer cells, as assessed by RT-PCR. The existence of very small, but functionally important, amounts of MMP-2 or – 8 however cannot be excluded unequivocally by the expression studies. MMP-1 is expressed by the human breast cancer cells in this study and may contribute to plasmin-dependent fibrillar collagen dissolution.

Currently, little is known with regard to the physiological mechanisms by which MMPs undergo activation in intact cell systems [23]. However, a primary function of the uPA-uPAR couple is to focus the processing of the plasminogen zymogen to active plasmin on the cell surface. It has recently been demonstrated that binding of u-PA to its receptor increases pro-MMP-2,-9 [23] and -13 [16] activation, and that in the absence of cells, plasmin not only fails to activate pro-MMPs, but rapidly degrades them [23]. This would explain why interfering with only one element, plasmin or MMP activity, has such powerful biological effects. It should be stressed, however, that few of the cascades or activities have been unequivocally documented in intact cell systems. Nonetheless, because MMP-specific inhibitors are not yet available, the individual role of each of the cell-derived MMPs awaits the use of interventions based on RNA inhibition.

The increased bone resorption that accompanies breast cancer cell infiltration of bone may arise as a result of (1) breast cancer cells stimulating osteoblast and osteoclast activity and (2) the production by breast cancer cells of proteinases that participate in degrading the organic aspect of bone matrix which would also facilitate the access of osteoclasts to the underlying mineralized matrix.

Even if tumour-derived MMPs do not directly digest mineralized matrix, they may participate in osteolysis via a mechanism that is analogous to a known osteoblast function. It is believed that all endosteal surfaces are covered with a layer of nonmineralized matrix. In areas of bone formation, this layer is called osteoid and it is easily observed in stained sections [24]. Evidence is accumulating that degradation of this layer must occur before the attachment of osteoclasts to the underlying mineralized matrix [25]. In normal remodelling, digestion of this layer is apparently accomplished by MMPs produced by osteoblasts [25-27]. It is conceivable that breast cancer cells in bone adopt this osteoblast function; thus, the possibility that MMPs produced by cancer cells enhance osteoclastic degradation by prior removal of the overlying unmineralized layer. Furthermore, removal of this layer may be partially responsible for the recruitment and activity of osteoclasts due to the release of osteoclast attractants/stimulants. Bone resorption by osteoclasts is augmented experimentally by coating mineralized matrix with collagenase-cleaved collagen fragments [28]. As nonmineralized matrix is degraded, osteoclasts may be exposed to extracellular proteins, such as fibronectin, vitronectin, osteopontin or other cryptic epitopes. Osteoclasts bind to these proteins via integrins a process that may enhance bone resorption [29].

## Conclusion

In conclusion, we have shown that breast cancer cells can degrade the organic aspect of bone matrix in contrast to non-tumourogenic breast epithelial cells. There is an absolute requirement for both the PA system and MMPs in the degradation process.

## Materials and methods

### Reagents

The MMP inhibitor CT-1166 was a generous gift from Dr A Docherty, Celltech (Slough, UK). The PAS inhibitor aprotinin, plasminogen and TGFβ were purchased from Sigma. The human mammary epithelial cell (HMEC) line, Mammary Epithelial Basal Medium (MEBM), insulin, hydrocortisone, gentamicin/amphotericin-B and Bovine Pituitary Extract (BPE) were purchased from Clonetics (Walkersville, MD, USA). Human breast tumour cell lines MDA-231, ZR-75-1 and MCF-7 were purchased from ATCC (American Type Culture Collection, Manassas, VA, USA). Gelatinase-A, TIMP-1 and polyclonal sheep antibodies to human MMP-1, -2,- 3, -9 and -13 were gifts from Dr J Reynolds, Strangeways Research Laboratory, Cambridge, UK. Neutralizing mAbs against human uPA or human uPAR were from American Diagnostica, Greenwich, CT, USA.

### Cell cultures

Cell lines were cultured in 75-cm^2 ^plastic tissue culture flasks (Costar Corning, Cambridge, MA, USA) as follows: ZR-75-1, MDA-231, MCF-7 and MC-3T3-E1 in α-MEM and HME cells in MEBM medium. All media were supplemented with 10% heat inactivated fetal bovine serum (FBS), 2 mM L-glutamine, 100 U/ml penicillin and 100 ug/ml streptomycin (Sigma Chemical Co.). Cultures were maintained at 37°C in a humidified atmosphere of 95% air and 5% carbon dioxide and subcultured every third day.

### Type I collagen degradation assay

^14^C-labelled collagen films were prepared as described previously [30]. Breast cells were seeded into collagen coated 16 mm tissue culture wells at a density of 1 × 10^5 ^/well. After 4 h incubation at 37°C in MEM supplemented with 10% FCS, the cells were washed twice with phosphate buffered saline (PBS) to remove residual FCS, and incubated for 24 h with 1 ml/well of serum-free MEM with or without the indicated concentrations of plasminogen, TGFβ and the proteinase inhibitors to be tested. At the end of the culture period the media were centrifuged (15 min, 1200 × g) to remove any collagen fibrils, and radioactivity released during collagen degradation quantified by liquid scintillation counting. Residual collagen was digested with bacterial collagenase (50 μg/ml) and assayed for radioactivity. Collagenolysis was expressed as radioactivity released from the films as a percentage of the total ± SEM.

### Formation of ^3^H-labelled, non-mineralized and mineralized bone matrix

The murine calvarial-derived MC3T3-E1 is a well characterized osteoblast culture system providing a suitable model of osteogenesis analogous to *in vivo *bone formation [15]. The cultures were maintained at 37°C in a humidified atmosphere of 95% air and 5% carbon dioxide. Culture medium was changed on the first day after seeding and then every 72 h. The cells were split after 7–9 days in culture, before they reached confluence, and plated at a density of 1 × 10^4 ^cells/well on collagen-coated 24-well plates (Becton Dickinson, MA, USA). After 4–5 days, when the cultures had reached confluence and the formation of an ECM had started, fresh medium was added containing 1 μCi/ml ^3^H amino acid mixture for 7 days (Amersham International, Aylesbury, UK) to create a non-mineralized radiolabelled ECM. In order to create a mineralized bone matrix, the medium was supplemented with 10 mM β-glycerol phosphate and the cells cultured for 14 days and the radiolabelled medium changed every 3 days. Unincorporated ^3^H-radiolabelled amino acids were washed from the remaining ECM using 2 water washes and 75% (v/v) ethanol. The matrices were dried and stored at -20°C until use.

### Von Kossa staining for mineralization

Mineralization of matrices was determined by von Kossa staining. The matrices were rinsed with cold PBS and fixed in 10% neutral buffered formalin for 15 min, rinsed with distilled water and left in distilled water for 15 min. Matrices were stained with 2.5 % silver nitrate solution for 30 min at room temperature. The silver nitrate was removed and matrices were rinsed with distilled water before the addition of sodium carbonate formaldehyde for 5 min. After rinsing, the matrices were counterstained with toluidine blue, rinsed with tap water and air dried.

### Bone matrix degradation assay

Breast cancer cells (10^5 ^/16 mm culture well) were seeded onto the bone matrix as for the type I collagen degradation assay. After 4 h incubation at 37°C in MEM supplemented with 10% FCS, the cells were washed twice with phosphate buffered saline (PBS) to remove residual FCS, and incubated for 24 h with 1 ml/well of serum-free MEM with or without the indicated concentrations of plasminogen, TGFβ and the proteinase inhibitors that were tested. After 24 h incubation, the media were removed and the extent of degraded ^3^H-radiolabelled matrix released into the medium was determined by liquid scintillation counting. The remaining matrix was degraded as for the type I collagen assay and matrix degradation expressed as above.

### PCR and RT-PCR

Confluent breast tumour cells were stimulated with TGFβ (10^-10 ^M) in serum-free medium for 24 h. For PCR analysis, oligonucleotide primers were synthesized based on the published sequences for 14 MMPs (i.e., MMP-1, -2, -3, -7,-8,-9,-10, -11, -12, -13,-14,-15, -16, and -17) [4]. t-PA, u-PA and, u-PAR oligonucleotides were designed using Designer PCR (Research Genetics, AL, USA) and primers purchased from Life Technologies Ltd. The housekeeping gene GAPDH was used as a positive control for the RT-PCR methodology.

Enzymes and buffers for the reverse transcriptase and PCR reactions were obtained from Perkin Elmer (CA, USA). Reverse transcriptase reactions were done according to the manufacturer's protocol using 1 μg of total RNA collected from untreated or TGF-β treated breast cells. PCR reactions were performed in an automated DNA thermal cycler (Perkin Elmer) for 25 cycles of denaturation (95°C, 1 min), annealing (variable time and temperature) and polymerisation (60°C, variable time). Amplification of the house-keeping gene, G3PDH mRNA (35 cycles), provided an internal control for the efficiency of the RT-PCR process. RT-PCR products were analyzed against molecular weight standards (pBR322 HaeIII digest) on a 2.5% agarose gel stained with ethidium bromide, electrophoresed in 0.5× TBE buffer at 100 V for 90 minutes. Gels were examined under ultraviolet light and photographed. The authenticity of the PCR products was verified by sequencing [31].

### Western Blot analysis

To identify MMP proteins and uPA present in breast cell conditioned medium, Western blot analysis was performed using their specific antibodies as previously described [32]. Briefly, samples were separated by 8.5% SDS-PAGE, transblotted on to PVDF membranes (Millipore Corp., MA, USA) and immunoblotted with polyclonal sheep antiserum to MMPs or polyclonal goat antiserum to uPA and secondary peroxidase-conjugated anti-sheep / anti-goat antibodies. Labelled proteins were detected with ECL detection solution and exposed to autoradiographic film (Hyperfilm ECL, Amersham International, UK).

### Assay of collagenase activity

To measure collagenase activity, TGFβ (10^-10 ^M)-stimulated breast cells were cultured in the presence/absence of 2 ug/ml of human plasminogen for 24 h. Collagenase activity was measured by the degradation of fluorescein isothiocyanate (FITC)-labelled type I collagen using type I activity assay kits. One unit of these activities degrades 1 μg of collagen per min at 37°C.

### uPA Assay

Cells were plated in 6-well trays and grown to near confluence, the medium was removed, and the cells were washed and incubated with 2 ml of serum-free medium for 24 hr. The medium was collected, centrifuged, and frozen until assayed. The cells were lysed in 300 ul of 0.1 M Tris (pH 8.1) and 0.1% Triton X-100. The u-PA (10 ul of conditioned medium and 10 μg of cell protein) was assayed as previously described [41] using plasminogen and S2251, the chromogenic substrate for plasmin.

### Statistical Analysis

Differences between control and treatment groups were determined by the Mann Whitney U-test.
